# Molecular Cloning and Functional Characterization of a Sterol 3-*O*-Glucosyltransferase Involved in Biosynthesis of Steroidal Saponins in *Trigonella foenum-graecum*

**DOI:** 10.3389/fpls.2021.809579

**Published:** 2021-12-13

**Authors:** Jianghong Gao, Yehan Xu, Congkun Hua, Changfu Li, Yansheng Zhang

**Affiliations:** Shanghai Key Laboratory of Bio-Energy Crops, Research Center for Natural Products, Plant Science Center, School of Life Sciences, Shanghai University, Shanghai, China

**Keywords:** saponin, sterol 3-*O*-glucosyltransferase, *Trigonella foenum-graecum*, dioscin, diosgenin

## Abstract

Fenugreek (*Trigonella foenum-graecum*), a pharmacologically important herb, is widely known for its antidiabetic, hypolipidemic, and anticancer effects. The medicinal properties of this herb are accredited to the presence of bioactive steroidal saponins with one or more sugar moieties linked to the C-3 OH position of disogenin or its C25-epimer yamogenin. Despite intensive studies regarding pharmacology and phytochemical profiles of this plant, enzymes and/or genes involved in synthesizing the glycosidic part of fenugreek steroidal saponins are still missing so far. This study reports the molecular cloning and functional characterization of a key sterol-specific glucosyltransferase, designated as TfS3GT2 here, from fenugreek plant. The recombinant TfS3GT2 was purified *via* expression in *Escherichia coli*, and biochemical characterization of the recombinant enzyme suggested its role in transferring a glucose group onto the C-3 hydroxyl group of diosgenin or yamogenin. The functional role of TfS3GT2 in the steroidal saponin biosynthesis was also demonstrated by suppressing the gene in the transgenic fenugreek hairy roots *via* the RNA interference (RNAi) approach. Down-regulation of TfS3GT2 in fenugreek generally led to reduced levels of diosgenin or yamogenin-derived steroidal saponins. Thus, Tf3SGT2 was identified as a steroid-specific UDP-glucose 3-*O*-glucosyltransferase that appears to be involved in steroidal saponin biosynthesis in *T. foenum-graecum*.

## Introduction

*Trigonella foenum-graecum* (fenugreek) is one of the oldest traditional medicinal plant species originating from the Iran and Mediterranean regions (Mostafaie et al., 2018). In ancient Egypt, fenugreek was documented for increasing milk production in lactating women and treating mummies (Nagulapalli Venkata et al., 2017). In traditional Chinese medicine, this plant has been prescribed for the treatment of many conditions, such as lung congestion, diabetes, epilepsy, and paralysis (Nagulapalli Venkata et al., 2017). Currently, various medicinal properties of fenugreek have been revealed, such as antidiabetic (Raju et al., 2001), antiobesity (Gao et al., 2015), anticancer (Shabbeer et al., 2009), and sex-promoting (Aswar et al., 2010) activities.

Phytochemical studies of fenugreek seeds and other tissues have revealed the presence of steroidal saponins primarily based on spirostanol aglycones (e.g., diosgenin or its C25-epimer yamogenin) (Pang et al., 2012; Kang et al., 2013; Krol-Kogus et al., 2020). The steroidal saponins are formed by successively attaching more than one sugar moieties onto the C3-hydroxy groups of the alycones, and the first linked sugar group is almost completely a D-glucose in β-configuration (Pang et al., 2012; Kang et al., 2013; Krol-Kogus et al., 2020). Among the steroidal saponins, dioscin has received increasing attentions, and since 1960s, in the former soviet, this compound was used as a main active component of herbal products to treat coronary heart diseases (Li et al., 2021). Dioscin is the major active ingredient of the traditional Chinese medicine product, called “Di’ao Xinxuekang capsule” (Yu et al., 2014), which is currently utilized for the prevention of cardiovascular diseases. Despite intensive studies concerning the chemical structures and medicinal activities of the saponins, the genes and enzymes involved in biosynthesis of the steroidal saponins in fenugreek remain largely unknown.

**FIGURE 1:**
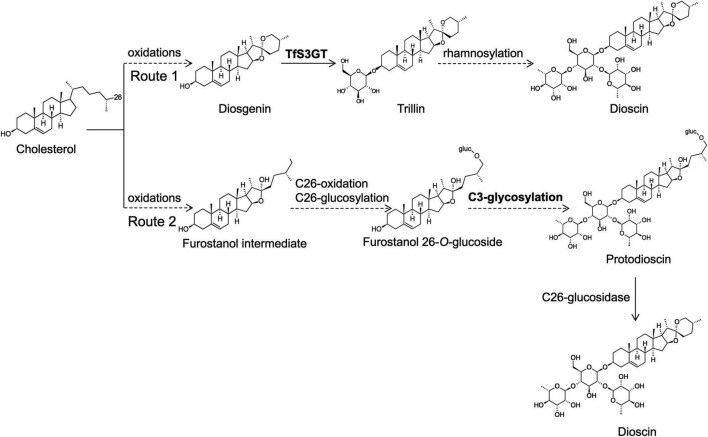
Proposed two glycosylation schemes that may be involved in dioscin biosynthesis in *T. foenum-graecum* plants. In the route 1, the glycosidic parts (one glucose and two rhamnose moieties) of dioscin are added after the formation of diosgenin skeleton. Diosgenin is biosynthesized from multiple oxidations of cholesterol catalyzed by cytochrome P450s (Christ et al., 2019). In the route 2, the glycosidic parts of dioscin are presumed to be introduced before the formation of diosgenin skeleton, and they may be added at the furostanol level. Cholesterol is converted to proto-dioscin in which the ring F is held open by C26-glucosylation, and from the proto-dioscin intermediate cleavage of the C26-glucose group would evoke closure of the ring F, ultimately producing dioscin.

Little is known about the biosynthetic steps leading to dioscin production in plants. The aglycone part of dioscin is diosgenin, and early labeling studies (Bennett and Heftmann, 1965; Joly et al., 1969b; Stohs et al., 1969) indicated that diosgenin is derived from cholesterol. Very recently, cytochrome P450s capable of converting cholesterol to diosgenin have been characterized from *T. foenum-graecum* and *Paris polyphylla* (Christ et al., 2019). Some researchers have proposed that dioscin is directly biosynthesized from diosgenin, simply by adding one glucose and two rhamnose groups at its C-3 OH position (see the route 1 of Figure 1; Ye et al., 2017; Li et al., 2018). However, this hypothesis is challenged by the natural occurrence of furostanol saponins (e.g., protodisocin, see its structure in Figure 1) in *T. foenum-graecum* (Kang et al., 2013; Krol-Kogus et al., 2020) and dioscorea genus (Li et al., 2010; Zhang et al., 2014). Unlike dioscin that bears the rings of E and F (see its structure in Figure 1), protodioscin lacks the ring F where the side chain is held open by C26-glucosylation (Figure 1). The widespread presence of protodioscin in the dioscin-producing species suggests that an alternative approach (see the route 2 of Figure 1) to biosynthesize dioscin occurs in nature. Indeed, Joly et al. ever provided direct evidence supporting a transformation of cholesterol to protodioscin in *Dioscorea floribunda* (Joly et al., 1969a). If the route 2 is followed, the addition of sugar groups must happen before the formation of diosgenin skeleton during dioscin biosynthesis. In either case, sterol glycosyltransferases (SGTs) responsible for the transfer of sugar groups during biosynthesis of steroidal saponins in *T. foenum-graecum* remain to be uncovered, as no relevant work has been made on this species.

In this study, we utilized a transcriptomics approach to screen putative SGTs involved in biosynthesis of the steroidal saponins from *T. foenum-graecum*. Biochemical characterization showed that one of the encoded enzymes (named TfS3GT2) is a steroid-specific 3-*O*-glucosyltransferase. This enzyme catalyzes the transfer of the first glucose group onto the C3-hydroxy group of diosgenin or yamogenin. The importance of this gene for steroidal saponin biosynthesis in *T. foenum-graecum* was revealed by silencing this gene using the RNA interference (RNA) approach. The probable stage (i.e., route 1 or 2 in Figure 1) at which TfS3GT2 catalyzes the glucosylation reaction is discussed.

## Materials and Methods

### Plant Materials and Chemicals

*Trigonella foenum-graecum* seeds from Shanxi Province of China were used in this study. The chemical standards of UDP-glucose, UDP-rhamnose, UDP-xylose, diosgenin, yamogenin, cholesterol, β-sitosterol, ergosterol, brassicasterol, campesterol, kaempferol, quercetin, myricetin, protopanoxadiol, ursolic acid, trillin, prosapogenin A (α-L-rhamnopyranosyl-(1 → 2)-β-D-glucopyranoside-3-*O*-diosgenin), zingiberoside A1 (α-L-rhamnopyranosyl-(1 → 2)-β-D-glucopyranoside-3-*O*-yamogenin), and dioscin (α-L-rhamnopyranosyl-(1 → 4)-[α-L-rhamnopyranosyl-(1 → 2)]-β-D-glucopyranoside-3-*O*-diosgenin) were purchased from Shanghai Yuanye Biotechnology Co., Ltd. (Shanghai, China). The ruscogenin and stigmasterol standards were ordered from Chengdu HerbPurify Biotechnology Co., Ltd. (Chengdu, China). High performance liquid chromatography (HPLC)-grade acetonitrile or methanol (Thermo Fisher Scientific, United States) was used for HPLC and liquid chromatograph-mass spectrometer (LC-MS) analysis.

### Isolation and Cloning of the Sterol C3-Glucosyltransferase Candidate Genes From *T. foenum-graecum*

Our previously reported *T. foenum-graecum* transcriptome (Zhou et al., 2019) was used for gene isolation. Five sterol C3-glucosyltransferase (S3GT) candidates (Cluster-2140.105632, Cluster-2140.95550, Cluster-2140.71031, Cluster-2140.131704, and Cluster-2140.319) were identified by a BLAST search against the known *Dioscorea zingiberensis* S3GTs (namely Dz3GT1 or Dz3GT2) that we previously have reported (Li et al., 2018). Except for the cluster-2140.95550, the other four gene candidates could be amplified by standard RT-PCR from the methyl jasmonate (MeJA)-treated *T. foenum-graecum* seedlings, which are the same set of plant materials that we previously used for establishing the *T. foenum-graecum* transcriptome (Zhou et al., 2019). The successfully amplified four candidates were designated as TfS3GT1 (cluster-2140.105632), TfS3GT2 (cluster-2140.71031), TfS3GT3 (cluster-2140.319), and TfS3GT4 (cluster-2140.131704), respectively, and they were subsequently cloned into an *Escherichia coli* expression vector pGEX-2T *via Bam*HI/*Eco*RI sites using a ClonExpress^®^ One Step Cloning Kit (Vazyme, China). The N-terminus of TfS3GT1-4 was designed to be fused in a frame with a glutathione-S-transferase (GST) tag present in the pGEX-2T vector.

### Preparation of Recombinant TfS3GTs and *in vitro* Enzyme Assays

The above resulting vectors were transformed into *E. coli* strain TSsetta DE3. The cells were incubated in a Lysogeny Broth (LB) medium supplemented with 100 μg/mL ampicillin and 50 μg/mL chloramphenicol at 37°C until A_600_ reached 0.6–0.8. After addition of IPTG (isopropyl-β-D-thiogalactoside) at a final concentration of 1 mM, the cell were further cultured at 16°C for 16 h, then harvested and re-suspended in a lysis buffer (10 mM KH2PO4 pH 8.0, 140 mM NaCl, 2.7 mM KCl, 10 mM Na2HPO4, 1 mM EDTA, 2 mM dithiothreitol). Following disruption of the cells by sonication, the soluble solution was loaded onto a column filled with GST-binding magnetic beads. Recombinant TfS3GTs were then purified with an elution buffer (0.1 M potassium phosphate pH 8.0, 10 mM reduced glutathione), and concentrated into a reaction buffer (50 mM Tris–HCl; pH8.0) through a 30 kDa-desalting filter. The purity of the recombinant TfS3GTs was monitored by SDS-PAGE (sodium dodecyl sulfate polyacrylamide gel electrophoresis) analysis, and protein concentrations were measured by the Bradford assays.

Unless otherwise stated, the *in vitro* assays (in a total volume 100 μL) consisted of 50 mM Tris–HCl buffer (pH 8.0), 100 mM DTT, 30 mM NADPH, 50 mM sugar donor (UDP-glucose, UDP-rhamnose, or UDP-xylose), 5 mM substrate, and 7 μg purified TfS3GT. The mixture was incubated overnight at 30°C, and extracted three times with 800 μL of ethyl acetate. The ethyl acetate extracts were evaporated to dryness, and re-dissolved in 100 μL methanol for HPLC or LC-MS analysis. To measure the reaction velocities of TfSGT2 or TfS3GT4, assays were carried out for 20 min, which was proven to be a linear reaction time by preliminary experiments. For each enzyme, the assays were performed in triplicate.

### Preparation of the *TfS3GT2* RNAi Construct and Generation of the Transgenic *T. foenum-graecum* Hairy Roots

To prepare the *TfS3GT2* RNAi construct, specific primers (see Supplementary Table 1) were designed to amplify a 483 bp-fragment from the *TfS3GT2* coding region. The amplified PCR products were first cloned into an intermediate vector pDONR201, and then introduced into a binary vector pK7GWIWG2_II-RedRoot to form a hairpin cassette using the Gateway LR recombination reaction (Thermo Fisher Scientific, United States). The vector “pK7GWIWG2_II-RedRoot” harbors a red fluorescence protein (RFP) as a selection marker. After confirmation of the construct by sequencing, the binary vector that contains the RNAi construct for *TfS3GT2*, as well as the empty vector, were transformed into the *Agrobacterium rhizogenes* ARqua1 strain (Boisson-Dernier et al., 2001) by electroporation, and then were used for infection of *T. foenum-graecum* seedlings. The protocol, as previously described by Garagounis et al. (2020), was used to generate the *T. foenum-graecum* hairy roots with a few modifications. In brief, 3 day-old seedlings after seed germination were subjected to the infection with the ARqual strain. After cutting off the seedling radicles, the traumatized radicle surface was soaked in the *Agrobacterium* slurry harboring the target constructs for 3–5 min. The inoculated seedlings were then cultured on vertically positioned square plates, which contain half-strength MS agar media, in a growth chamber at 22°C in a 16/8 h light/dark cycle.

About 2 weeks after the infection, calluses started to form on the infected sites, while hairy roots emerged about 3 weeks post the inoculation. About 40 days later, positive roots were confirmed by examining red fluorescence signals, which resulted from expression of the RFP marker present in the RNAi construct, using a fluorescence microscope (Nikon SMZ1500, Japan). The filter sets used for excitation (Ex) and emission (Em) were as follows: RFP, 561 nm (Ex)/from 579 to 675 nm (Em); bright field, 633 nm. Signals were captured in multi-channel mode. The positive hairy roots were frozen in liquid nitrogen, and stored at –80°C for gene expression analysis and metabolite measurement later. Each biological replicate contained the hairy roots that were pooled from 30 infected plants, and data for each construct was collected from at least three biological repeats.

### Metabolite Extraction From the *T. foenum-graecum* Hairy Roots

The hairy root samples were ground into a fine powder in liquid nitrogen, and were dried to constant weight in a 37°C-oven. To measure diosgenin content in the hairy root samples, the extraction was carried out as follows: 20 mg of each dried hairy root sample was extracted with 3 mL methanol, and the methanol extracts were evaporated to dryness, acid-hydrolyzed with 1.8 M sulfuric acid at 100°C for 10 h, and then extracted with 9 mL hexane. The hexane extracts were washed with water, evaporated to dryness, and then re-dissolved in 150 μL of methanol for HPLC analysis. For each sample, 60 μg of ursolic acid was included as an internal standard to normalize possible variations introduced by different extractions. To measure saponin contents in the hairy root samples, 20 mg of each dried samples was immersed in 1 mL of methanol overnight, then extracted in ultrasound bath for 1 h with addition of another 2 mL methanol. The methanol extracts were filtered through 0.22 μm nylon syringe prior to LC-MS analysis. The metabolite measurement was carried out in three biological replicates.

### High Performance Liquid Chromatography and Liquid Chromatograph-Mass Spectrometer Analysis

For HPLC analyses, samples were detected on a SIL-20A HPLC system (Shimadzu, Japan) equipped with an Extend-C18 column (250 mm × 4.6 mm, 5 μm) at 30°C. The UV detection wave length was set to 203 nm. For the products from *in vitro* enzyme assays with cholesterol, β-sitosterol, ergosterol, stigmasterol, brassicasterol or campesterol, milli-Q water (solvent A), and HPLC-grade methanol (solvent B) were used as the mobile phase, and samples were separated using 98% B at a flow rate of 1.0 mL/min for 40 min. For the products from the *in vitro* assays with diosgenin, yamogenin, ruscogenin, protopanoxadiol or ursolic acid, milli-Q water (solvent A), and HPLC-grade acetonitrile (solvent B) were used as the mobile phase, and samples were separated at a flow rate of 0.8 mL/min using 60–100% B for 35 min, 100% B for 5 min, 100–60% B for 2 min, and 60% B for 8 min. For the products from the *in vitro* enzyme assays with kaempferol, quercetin, myricetin, the mobile phase was composed of water with 0.1% formic acid (solvent A) and HPLC-grade acetonitrile (solvent B), and samples were separated at a flow rate of 0.8 mL/min using 10–85% B for 10 min, 85% B for 6 min, 85–10% B for 6 min, and 10% B for 4 min. To measure diosgenin contents in the transgenic hairy roots, the mobile phase was composed of 0.1% phosphoric acid (solvent A) and 70% methanol/30% acetonitrile (solvent B), and samples were separated using 90% B for 45 min at a flow rate of 0.8 mL/min.

To monitor accumulation of the saponins in the hairy roots, LC-MS analysis was performed using a Q-Exactive Focus mass spectrometers, coupled with a VanquishTM UPLC system (Thermo Fisher Scientific, United States) and a HESI source (Thermo Fisher Scientific, United States). The column (100 mm × 2.1 mm, 1.8 μm) was used to separate the sample, the column temperature was 45°C, and the flow rate was 600 μL/min. The mobile phases contain 0.1% formic acid (solvent A) and acetonitrile (solvent B), and the solvent gradient is set as follows: 20–22% B for 1 min, 22% B for 3 min, 22–24% B for 11 min, 24–30% B for 0.1 min, 30–40% B for 4.4 min, 40–44% B for 2 min, 44–60% for 7.5 min, 60–100% B for 1 min, 100% B at 2 min. The ESI-Q-Exactive Orbitrap mass spectrometer (Thermo Fisher Scientific, MA, United States) was operated in a full-scan and positive mode under the following conditions: resolution of 70,000; scan range of 100.0–1500.0 m/z; polarity is positive; AGC target at 3 × 10^6^; maximum inject time is 120 ms. The parameter setting conditions of HESI source are as follows: sheath gas flow rate, aux gas flow rate and sweep gas flow rate are 40, 15, and 1, respectively; spray voltage is 3.00 kV; capillary temperature at 275°C; s-lens RF level at 55.0; aux gas heater temperature is 310°C. The instrument was controlled by Xcalibur software (Thermo Fisher Scientific, United States).

### Quantitative Real-Time PCR

Total RNA was extracted from the transgenic hairy roots using an EASYspin Plus Plant RNA Extraction Kit (Aidlab, China). The first-strand cDNA was synthesized using the TransScript One-Step gDNA Removal and cDNA Synthesis SuperMix Kit (TransGen Biotech, China). Quantitative RT-PCR (qRT-PCR) was performed on a ABI 7500 Fast Real-Time PCR Detection System with TOROGreen^®^5G qPCR PreMix Kit (TOROIVD, China). The PCR conditions were set as follows: 10 min of initial denaturation at 95°C, followed by 40 cycles of 95°C for 20 s, and then 60°C for 1 min. All real-time PCR was performed in three independent repeats. The primers used for the qRT-PCRs are listed in Supplementary Table 1.

### Statistical Analysis

Every experiment was carried out at least in three biological replicates, and data were shown as mean ± SD. Data analysis was performed by one-way ANOVA. Difference was considered statistically significant when *p* < 0.05 (**), and extreme significant when *p* < 0.01 (***).

## Results

### Phylogenetic Analysis and Functional Characterization of the TfS3GT Candidates

#### Phylogenetic Analysis

**FIGURE 2:**
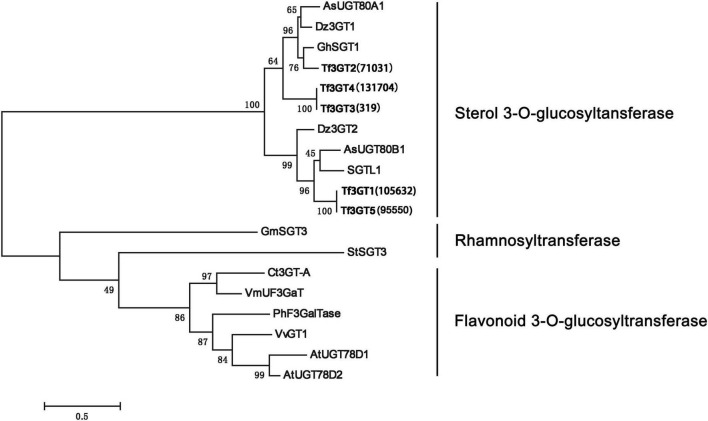
Phylogenetic analysis of the four TfS3GTs together with the previously characterized glucose- or rhamnose- transferases. The evolution history was inferred based on the neighbor-joining method using the software MEGA 7.0 (Kumar et al., 2016). The analysis incudes 14 previously published sequences, and their Genbank accession numbers are listed in the Supplementary Table 2. The bootstrap values are 500, and the scale bar represents 0.5 amino acid substitutions per site.

A phylogenetic analysis of the TfS3GT candidates and some previously published 3-*O*-glucosyltransferases and rhamnosyltransferases (see their GenBank accession numbers in Supplementary Table 2) was performed (Figure 2). The TfS3GT1-4 candidates all clustered into the clade composed of sterol-3-*O*-glucosyltransferases, while the flavonoid 3-*O*-glucosyltransferases formed a distinct clade in the tree, suggesting that the TfS3GT candidates are relatively more related to sterol 3-*O*-glucosyltransferases.

#### Functional Characterization of the TfS3GT Candidates

**FIGURE 3:**
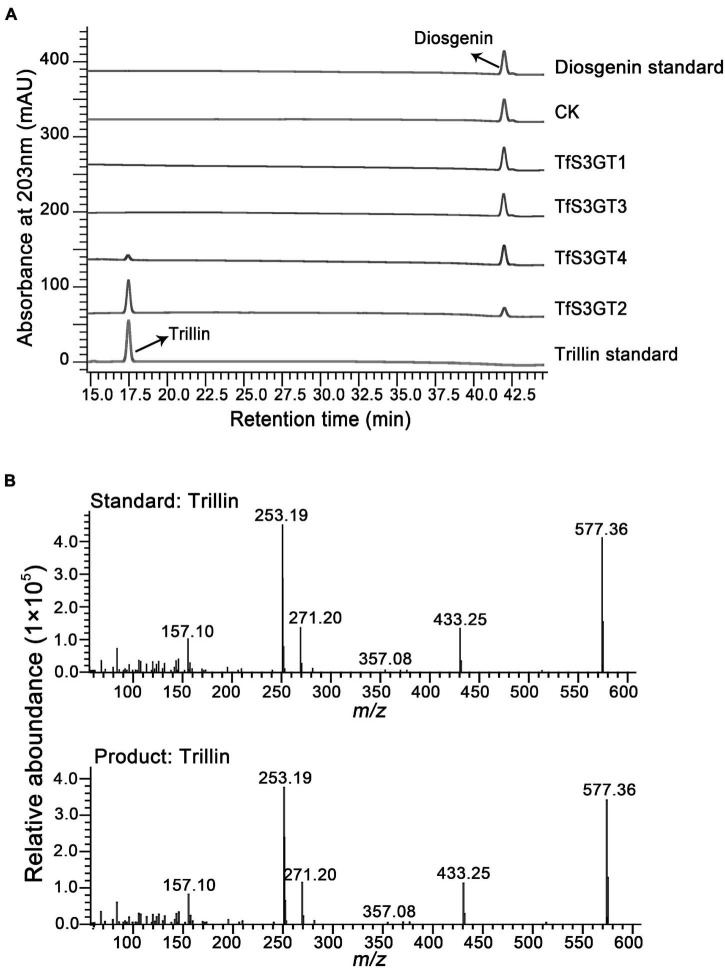
HPLC analysis of the products extracted from the *in vivo* assays of TfS3GT2 or TfS3GT4 with diosgenin **(A)**. The mass spectrum of the trillin product in comparison with the trillin standard **(B)**.

To characterize the catalytic functions of the TfS3GTs *in vitro*, recombinant TfS3GTs were expressed in *E. coli* cells, and purified by GST-tag affinity chromatography (Supplementary Figure 1). The *in vitro* assays were first performed using diosgenin as the sugar acceptor and uridine 5′-diphosphate glucose (UDP-Glc) as the sugar donor. The reaction products were then subjected to HPLC or LC-MS analysis. The negative control reaction was performed by omitting the recombinant TfS3GTs in the reaction mixture. Among the four TfS3GT candidates, only TfS3GT2 and TfS3GT4 showed an activity of converting diosgenin to a new product (Figure 3A). This product showed the same retention time and mass spectrum as authentic trillin (i.e., diosgenin-3-*O*-glucoside) (Figure 3B), supporting that either TfS3GT2 or TfS3GT4 is a functional sterol-3-*O*-glucosyltransferase that catalyzes the formation of trillin from diosgenin. By performing the assays in a linear reaction time of 20 min, the reaction velocities of TfS3GT2 and TfS3GT4 with diosgenin were calculated to be 6.50 ± 0.10 and 0.88 ± 0.29 nM/min/mg, respectively, suggesting that TfS3GT2 has a greater sterol-3-*O*-glucosyltransferase activity than TfS3GT4.

**FIGURE 4:**
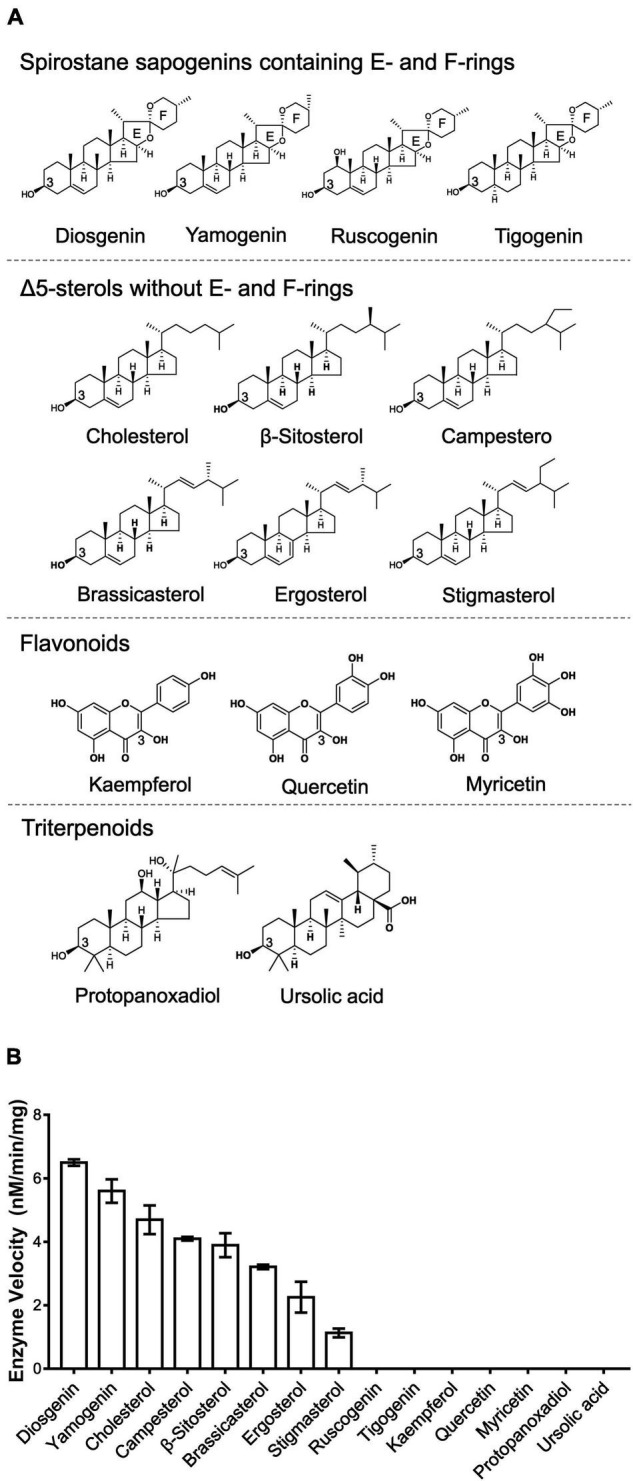
Enzyme activities of TfS3GT2 with different types of aglycones. **(A)** Chemical structures of four types of the alycones tested. **(B)** Enzyme activities of TfS3GT2 on four types of the aglycones with UDP-glucose as a sugar donor. In the *in vitro* enzyme assays, saturate concentrations of the acceptors and the UDP-glucose donor were utilized (see the section “Materials and Methods”). The assays were carried out for 20 min. The enzyme activities of TfS3GT2 on various alycones were quantified based on the mole numbers of the aglycones consumed by the enzyme, relative to the control reactions which do not include TfS3GT2. Values represent the means ± SD from three independent assays.

To explore the sugar donor promiscuity of TfS3GT2 or TfS3GT4, UDP-xylose and UDP-rhamnose were supplied in the *in vitro* assays, using diosgenin as an acceptor. The results demonstrated that both TfS3GT2 and TfS3GT4 could not accept UDP-xylose or UDP-rhamnose as a sugar donor. Considering that TfS3GT2 displays a much higher activity relative to TfS3GT4 (Figure 3A), only TfS3GT2 was further subjected to the substrate specificity analysis. The optimum pH for TfS3GT2 activity was revealed to be 8.0, which was measured by comparing the activity of TfS3GT2 with diosgenin as an acceptor and UDP-glucose as a sugar donor within the pH range of 7.0–9.0 (Supplementary Figure 2). Next, using Tris–HCl (pH 8.0) as a reaction buffer, we tested the activity of TfS3GT2 with four types of compounds, which all share a hydroxyl group at their C3-positions. They are spirostane sapogenins containing E- and F-rings (i.e., diosgenin, ruscogenin, tigogenin, and yamogenin), Δ5-sterols without E- and F-rings (i.e., cholesterol, β-sitosterol, ergosterol, stigmasterol, brassicasterol, and campesterol), flavonoids (i.e., kaempferol, quercetin, and myricetin), and triterpenoids (i.e., protopanoxadiol and ursolic acid) (see their structures in Figure 4A). TfS3GT2 showed activities toward the Δ5-sterols with or without the E- and F-rings, but it did not show glucosylation activity on triterpenoid and flavonoid substrates (Figure 4B), demonstrating that TfS3GT2 is a sterol-specific 3-*O*-glucosyltransferase. Generally, TfS3GT2 showed the highest activity toward spinostanol sterols with 11.5–71.3% of the activity being found with the selected Δ5-sterols without the E- and F-rings (Figure 4B). Interestingly, TfS3GT2 showed no activity toward ruscogenin and tigogenin. Tigogenin is a spirostanol sapogenin bearing the E- and F-rings, but it lacks a C5-C6 double bond (Figure 4A). Ruscogenin differs from diosgenin only by the presence of a hydroxyl group at C1-position (Figure 4A).

### Saponin and Diosgenin Content in the *TfS3GT2*_RNAi Transgenic Hairy Roots

**FIGURE 5:**
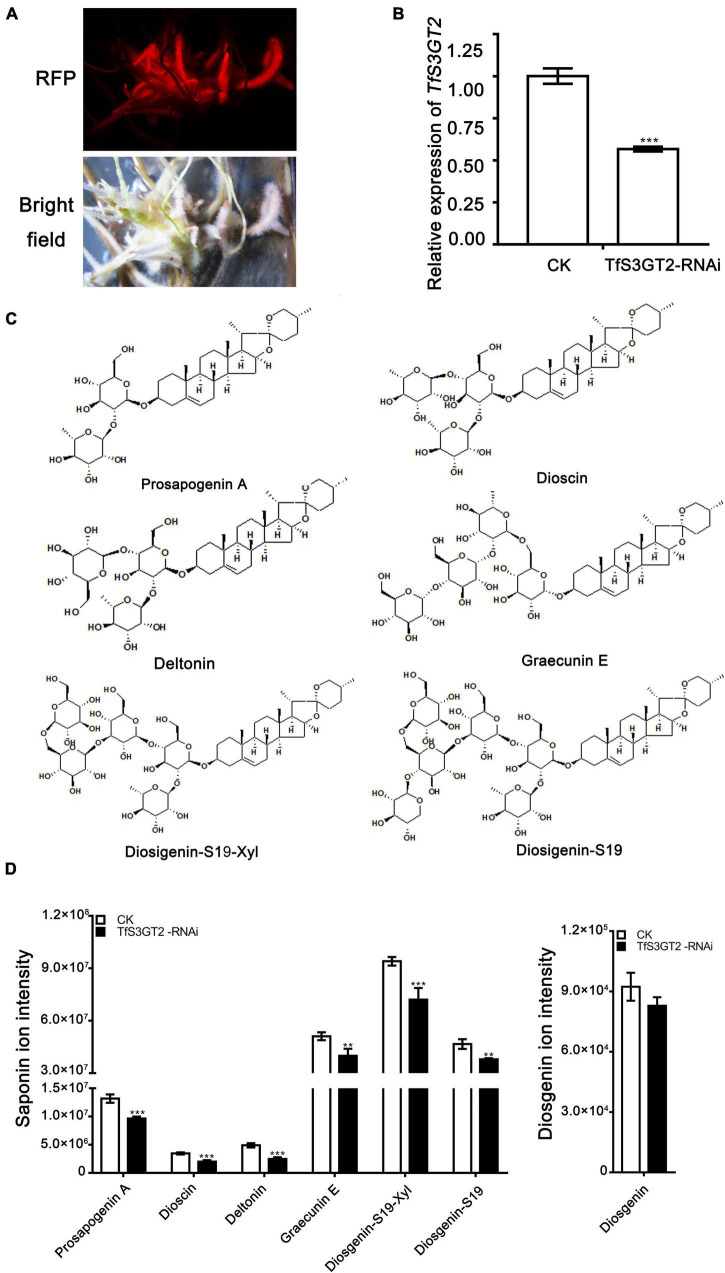
The effects of the RNAi-based silencing of *TfS3GT2* on biosyntheses of dioscin, diosgenin, and other targeted steroidal saponins in the transgenic *T. foenum-graecum* hairy roots. **(A)** Red fluorescence signal is shown for the presence of the TfS3GT2-RNAi construct in the hairy roots. Scale bar = 1 cm. **(B)** Real-time PCR analysis of the *TfS3GT2* transcript in the TfS3GT2-RNAi compared with the corresponding vector control lines. **(C)** Chemical structures of the steroidal saponins for the targeted analysis of the transgenic hairy roots. **(D)** The LC-MS product ion-intensities of the targeted steroidal saponins and diosgenin extracted from the transgenic hairy roots. The data are expressed as the mean ± SD of three biological replicates. Asterisk indicates a significant difference by one-way ANOVA analysis, and significant difference is indicated by ** when *P* < 0.05, and extremely significant difference is shown by *** when *P* < 0.01. The mass spectra of the targeted steroidal saponins are shown in Supplementary Figure 4.

In order to understand the *in planta* function of TfS3GT2, *T. foenum-graecum* seedlings were transformed with the *TfS3GT2*_RNAi construct to generate the transgenic hairy roots. Transformed roots were screened by red fluorescence examination under a microscope for the presence of the transgenes (Figure 5A). The expression level of *TfS3GT2* gene was analyzed by qRT-PCR in the *TfS3GT2*_RNAi and the vector control transformants. Expression of *TfS3GT2* gene was decreased up to 43.4% due to the silencing in comparison with the vector control (Figure 5B). To investigate the effects of the *TfS3GT2* silencing on the saponin and diosgenin contents in the transgenic hairy roots, quantification of six different diosgenin- or yamogenin-derived saponins (i.e., dioscin, prosapogenin A, (Figure 3A) deltonin, graecunin E, diosigenin-S19-Xyl, and diosigenin-S19) (see their structures in Figure 5C) was carried out using LC-MS analysis. Overall, with respect to the control, a significant decrease in the contents of all the diosgenin-derived saponins, with a percentage decrease of 18.7–45.9%, was observed (Figure 5D). Diosgenin content was slightly decreased, but the percentage decrease was not significant (Figure 5D).

## Discussion

This study pertains to be the first report on cloning and characterization of a steroid-specific metabolism gene (*TfS3GT2*) from *T. foenum-graecum*. The *TfS3GT2* mRNA was amplified only from the methyl-jasmonate (MeJA)-treated *T. foenum-graecum* seedlings, but not from the non-treated ones (data not shown), indicating that *TfS3GT2* mRNA was up-regulated by MeJA. This is consistent with the gene expression data acquired by our previously RNA-sequencing of *T. foenum-graecum* seedlings (Zhou et al., 2019), from which it appeared that *TfS3GT2* expression was strongly induced by MeJA. MeJA is a known signaling compound that can induce oxidative stress and biosynthesis of secondary metabolites in plant cells (Ho et al., 2020). Accordingly, at the beginning of this study, we speculated that *TfS3GT2* could be a defense gene leading to formation of some glycosides in response to oxidative stresses. In this study, biochemical and genetic functional analysis of TfS3GT2 demonstrate that TfS3GT2 involves in biosynthesis of dioscin and other diosgenin-derived glycosides. Interestingly, dioscin exerts its beneficial properties to human health, indeed largely *via* inhibition of oxidative stresses through different signaling pathways (Hu et al., 2018; Yang et al., 2018). TfS3GT2 also shows considerable activity in catalyzing glucosylation of several major plant-derived sterols (e.g., sitosterol, stimasterol, and campesterol) (Figure 3), further indicating its physiological role in adaptions to stressful environments, as glycosylation of the major plant sterols can provide increased tolerance to both abiotic and biotic stresses (Saema et al., 2016).

Our *in vitro* enzyme assays (Figure 4) revealed that TfS3GT2 showed strict substrate specificity with highly specific activity toward steroid aglycones, whereas non-steroidal substrates, including flavonoids and triterpenoids, were not active as sugar acceptors, strongly suggesting that TfS3GT2 is a sterol-specific glycosyltransferase. This biochemical finding well matched the result of the phylogenetic tree analysis, as in the tree TfS3GT2 showed a close relationship with sterol-3-*O*-glucosyltransferases but was distinct from others (Figure 2). The presence of a C5-C6 double bond in the steroid molecule seemed to be critical in glucosylation, as TfS3GT2 showed no detectable activity on tigogenin (Figure 4), which only differs from the best substrate diosgenin by the absence of a C5-C6 bond. Moreover, the selected triterpenoid substrates, which actually resemble the backbone structure of steroidal aglycones but lack a C5-C6 double bond, were also not glycosylated by TfS3GT2 at all (Figure 4). The presence of a C5-C6 double bond in steroid molecules conferring the substrate specificity was also demonstrated for a S3GT from *Withania somnifera* (Madina et al., 2007). Among the steroid substrates tested, diosgenin was the best aglycone substrate of TfS3GT2. To the best of our knowledge, in a pure form or as a crude extract, UGTs capable of catalyzing 3-*O*-glucosylation of diosgenin or yamogenin were only isolated from a few species, including *Solanum melongena* L (Pazkowski et al., 2001; Potocka and Zimowski, 2008), *W. somnifera* (Madina et al., 2007), and *D. zingiberensis* (Ye et al., 2017). In comparison with these previously reported S3GTs, TfS3GT2 was judged to be a novel diosgenin 3-*O*-glucosyltransferase based on the following observations: (1) The previously reported diosgenin 3-*O*-glucosyltransferases all give low activity on diosgenin, however, which is the best substrate here for TfS3GT2; (2) TfS3GT2 shares relatively low amino acid sequence identity (50–67%) with the previously reported diosgenin 3-*O*-glucosyltransferases. Interestingly, TfS3GT2 was not active with ruscogenin. It should be noted that ruscogenin has the C5-C6 double bond, and differs from diosgenin only by the presence of a hydroxyl group at C1-position (Figure 4A). The C1-OH of ruscogenin would increase the hydrophilicity, probably preventing it to be accommodated by TfS3GT2, since hydrophobicity is a common feature for the acceptor binding pocket of a sterol-specific glycosyltransferase (Chen et al., 2018). Indeed, diosgenin- or yamogenin-derived glycosides widely occur in *T. foenum-graecum*, but none of ruscogenin-derived saponins have been reported from this plant (Pang et al., 2012; Kang et al., 2013). To determine the substrate specificity of TfS3GT2 with respect to the sugar donor, UDP-glucose, UDP-xylose and UDP-rhamnose were tested, because they represent the dominant sugar moieties of steroidal saponins of *T. foenum-graecum* (Pang et al., 2012; Kang et al., 2013; Krol-Kogus et al., 2020). Only UDP-glucose was active as a sugar donor, whereas UDP-xylose or UDP-rhamnose could not replace UDP-glucose to facilitate the TfS3GT2 activity. The sugar donor specificity of TfS3GT2 is consistent with the fact that the first sugar moiety attached to the steroidal aglycones of *T. foenum-graecum* is almost completely the glucose group (Pang et al., 2012; Kang et al., 2013; Krol-Kogus et al., 2020).

In addition to TfS3GT2, this study has revealed another enzyme, designated as TfS3GT4, that also catalyzed the formation of diosgenin-3-*O*-glucoside (i.e., trillin) from disogenin (Figure 2). TfS3GT4 was calculated to exhibit only about 13.3% catalytic activity of TfS3GT2 in glycosylating the aglycone diosgenin. TfS3GT4 shares low sequence identity (54.4%) with TfS3GT2, with its N-terminal domain being shorter by 130 amino acid residues relative to TfS3GT2 (Supplementary Figure 3). The extremely low activity of TfS3GT4 toward diosgenin indicates that TfS3GT4 may have a unique substrate utilization, and diosgenin may not be its natural substrate *in vivo*. Recently, structural basis for the substrate specificity of a sterol 3β-glucosyltransferase, UGT51from *Saccharomyces cerevisiae*, has been elucidated (Chen et al., 2018). The yeast UGT51 shows considerable activities against ergosterol, cholesterol, and sitosterol (Warnecke et al., 1999), all of which can also be accepted as good substrates by TfS3GT2 (Figure 4). These observations tempted us to speculate that TfS3GT2 and UGT51 may share a similar structural mechanism for the activity, although there is only 14% amino acid identity between TfS3GT2 and UGT51 (Supplementary Figure 3). Amino acid sequence alignment showed that the nucleotide base binding motifs at the C-terminal domains of TfS3GT2, TfS3GT4, and UGT51 are highly conserved (Supplementary Figure 3), which is in agreement with the fact that they all display the same sugar donor specificity for the glucose. When focused on the acceptor-interacting residues in the cavity of UGT51 (Chen et al., 2018), TfS3GT2 and TfS3GT4 share the same residues at the equivalent positions with only one variable sequence at the 265 position (numbering in TfS3GT2) (Supplementary Figure 3). By contrast, the acceptor-interacting residues in UGT51 are distinct from the counterparts of TfS3GT2 or TfS3GT4 (Supplementary Figure 3), indicating that the SGTs from different organisms have evolved to accommodate diversely sterol acceptors present in their hosts, through changing only several residues within the active site of the enzyme.

This study could not provide conclusive information of which glycosylation scheme (i.e., routes 1 and 2 of Figure 1) that would be the major route for dioscin biosynthesis in *T. foenum-graecum*. Down-regulation of the *TfS3GT2* expression by our RNAi experiments led to a significant reduction in levels of dioscin and other diosgenin-derived saponins (Figure 5), strongly demonstrating that TfS3GT2 is involved in biosynthesis of steroidal saponins in *T. foenum-graecum*. If the sugar moieties are added after the formation of diosgenin, following the route 1 as shown in Figure 1, the reduction of the glycosides would in turn stimulate biosynthesis of the alycone backbone (i.e., diosgenin) through a feedback regulation. Through RNAi silencing of a UDP-glycosyltransferase, this type of the feedback regulation has also been observed for biosynthesis of withaferin A-derived saponins in *W. somnifera* (Saema et al., 2015) and ginsenosides in *Panax ginseng* (Lu et al., 2017). However, our data do not support this premise, as the aglycone diosgenin was not increased, and even was slightly decreased in the *TfS3GT2*-silenced lines (Figure 5). By contrast, if the sugar moieties are introduced before the formation of diosgenin skeleton following the route 2, the RNAi silencing of the first 3-*O*-glucosyltransferase would result in reduction in levels of both the saponins and diosgenin. Thus, the route 2 seems to give a plausible explanation of the data obtained from the RNAi experiments of this study, as the RNAi silencing of *TfS3GT2* indeed led to reduced levels of both diosgenin and its derived saponins, although the decrease in diosgenin was not statistically significant (Figure 5). One might argue that, in the biochemical assays of this study, diosgenin was revealed to be the best substrate of TfS3GT2 among the steroid substrates tested, thus favoring the route 1 that may exist in *T. foenum-graecum*. However, this puzzle could be explained by the catalytic plasticity of TfS3GT2 for different sterol substrates, which also seems to be a common feature of other previously reported sterol-glycosyltransferases (Pazkowski et al., 2001; Potocka and Zimowski, 2008; Stucky et al., 2015). Of course, this open scientific question deserves further investigations, which could be carried out by feeding radio-labeled glucose to the *TfS3GT2*-silenced or overexpressing lines, followed with phytochemical measurement of the radio-labeled glycosides.

## Conclusion

This study reports the isolation and functional characterization of a novel sterol-specific 3-*O*-glucosyltransferase (TfS3GT2) that is associated with the steroidal saponin biosynthesis in *T. foenum-graecum*. First, the recombinant TfS3GT2 was purified *via* expression in *E. coli*, and *in vitro* enzyme assays showed that TfS3GT2 exhibits considerable activities toward spinostanol sapongenins (including diosgenin and yamogenin) and other Δ5-sterols, whereas it is not active with other substrates, such as triterpenoids and flavonoids, supporting that TfS3GT2 is a sterol-specific glucosyltransferase. Next, we prepared the transgenic *T. foenum-graecum* hairy roots that decreased the *TfS3GT2* expression by the RNA interference approach. The RNAi silencing of TfS3GT2 led to a significant reduction in several steroidal saponins tested, including dioscin, strongly suggesting that TfS3GT2 is involved in biosynthesis of the steroidal saponins in *T. foenum-graecum*. We discussed the glycosylation scheme by which TfS3GT2 plays its catalytic role during dioscin biosynthesis *in vivo*.

## Data Availability Statement

The datasets presented in this study can be found in online repositories. The names of the repository/repositories and accession number(s) can be found in the article/Supplementary Material.

## Author Contributions

YZ designed the experiment. YZ and JG contributed to the writing. JG, YX, and CH performed the experiments. CL supervised the project and analyzed the data. All authors read and confirmed its content.

## Conflict of Interest

The authors declare that the research was conducted in the absence of any commercial or financial relationships that could be construed as a potential conflict of interest.

## Publisher’s Note

All claims expressed in this article are solely those of the authors and do not necessarily represent those of their affiliated organizations, or those of the publisher, the editors and the reviewers. Any product that may be evaluated in this article, or claim that may be made by its manufacturer, is not guaranteed or endorsed by the publisher.
