# Supplementary material for: Molecular Cloning and Functional Characterization of a Sterol 3-O-Glucosyltransferase Involved in Biosynthesis of Steroidal Saponins in Trigonella foenum-graecum
Source: Front Plant Sci. 2021 Dec 13;12:809579. doi: 10.3389/fpls.2021.809579 (PMC8710529; doi:10.3389/fpls.2021.809579)
Supplement: Supplementary file 1 [file Data_Sheet_1.PDF]

Supplementary Material

Molecular cloning and functional characterization of a sterol 3-*O*-glucosyltransferase involved in biosynthesis of steroidal saponins in *Trigonella foenum-graecum*

Jianghong Gao, Yehan Xu, Congkun Hua, Changfu Li*, Yansheng Zhang*

Shanghai Key Laboratory of Bio-Energy Crops, Research Center for Natural Products, Plant Science Center, School of Life Sciences, Shanghai University, Shanghai, China

*** Correspondence:**
Yansheng Zhang, zhangys1@shu.edu.cn
Changfu Li, changfuli@shu.edu.cn

Supplemental Tables

Supplementary Table 1. Primers used in this study

Primer name	Sequence (5' to 3')	Description
TfS3GT1-F	GATCTGGTTCCGCGTGGATCCATGATTTCAAGATCAATGACAGAAAAGAGAG	For expression in *E.coli*
TfS3GT1-R	TCAGTCAGTCACGATGAATTCCTACACACCACCACAAGGTAAAC	
TfS3GT2-F	GATCTGGTTCCGCGTGGATCCATGGCGGAATTGCCAGAGA	
TfS3GT2-R	TCAGTCAGTCACGATGAATTCTCAGGAACAACCAAAACATCTACGT	
TfS3GT3-F	GATCTGGTTCCGCGTGGATCCATGGCGAGTAATAATAATCATGACCG	
TfS3GT3-R	TCAGTCAGTCACGATGAATTCTTATCCAGAAGAATGGTAGCAACCAAAACA	
TfS3GT4-F	GATCTGGTTCCGCGTGGATCCATGGTTAAGGATGACGGTACTGTAG	
TfS3GT4-R	TCAGTCAGTCACGATGAATTCTTATCCAGAAGAATGGTAGCAACCAAAACA	
RNAi-TfS3GT2-F	GGGGACAAGTTTGTACAAAAAAGCAGGCTCCCTGGTAAAATGGCTTGAAGATG	To prepare the plant RNAi construct
RNAi-TfS3GT2-R	GGGGACCACTTTGTACAAGAAAGCTGGGTCCTCATTTTCCATGGCCTTTGC	
RT-TfS3GT2-F	TTTTCGCTTCCTAAGTTGATTGATG	For the qRT-PCR
RT-TfS3GT2-R	CGTATAGAGAAAAAACTAGATGGAGACG	
Actin-F	TCGCTGCTGAGGTTTTGGAA	
Actin-R	CCAATTTCGCCTTTGCCCTT	

Supplementary Table 2. The previously characterized glycosyltransferases used for the phylogenetic analysis of this study

Protein	GenBank accession Number	Plant species	Function
UGT80A1	CAB06081	*Avena sativa*	Sterol 3-*O*-glucosyltransferase
UGT80B1	OAP13743	*Arabidopsis thaliana*	Sterol 3-*O*-glucosyltransferase
GhSGT1	AHX00584	*Gossypium hirsutum*	Sterol 3-*O*-glucosyltransferase
SGTL1	ABC96116	*Withania somnifera*	Sterol 3-*O*-glucosyltransferase
Dz3GT1	AVI57699	*Dioscorea zingiberensis*	Sterol 3-*O*-glucosyltransferase
Dz3GT2	AVI57700	*D. zingiberensis*	Sterol 3-*O*-glucosyltransferase
GmSGT3	NP_001240857	*Glycine max*	soyasaponin III rhamnosyltransferase
StSGT3	NP_001274799	*Solanum tuberosum*	β-solanine/β-chaconine rhamnosyltransferase
Ct3GT-A	BAF49297	*Clitoria ternatea*	anthocyanidin 3-O-glucosyltransferase
UF3GaT	BAA36972	*Vigna mungo*	flavonoid 3-O-galactosyl transferase
F3GalTase	AAD55985	*Petunia hybrida*	flavonol 3-O-galactosyltransferase
UGT78D1	AAF19756	*Arabidopsis thaliana*	flavanoid 3-o-glucosyltransferase
VvGT1	AAB81683	*Vitis vinifera*	flavonoid 3-o-glucosyltransferase
UGT78D2	CAC01718	*Arabidopsis thaliana*	flavonoid 3-o-glucosyltransferase

Supplemental Figures

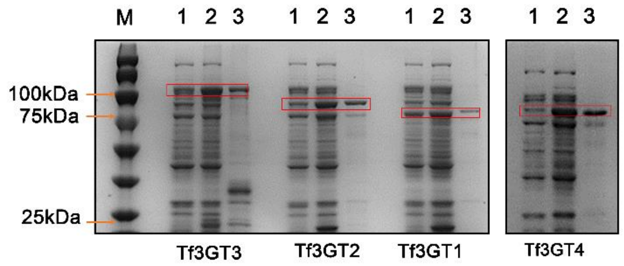

Supplementary Figure 1. SDS-PAGE analysis of the purified recombinant TfS3GT1, TfS3GT2, TfS3GT3 and TfS3GT4. M, protein molecular marker; Lane 1, the protein extract from the non-inducting culture without addition of the inducer IPTG; Lane 2, the protein extract from the inducing culture with addition of 1 mM IPTG; Lane 3, the purified recombinant TfS3GTs. The bands indicated by red square frames refer to the expressed TfS3GTs.

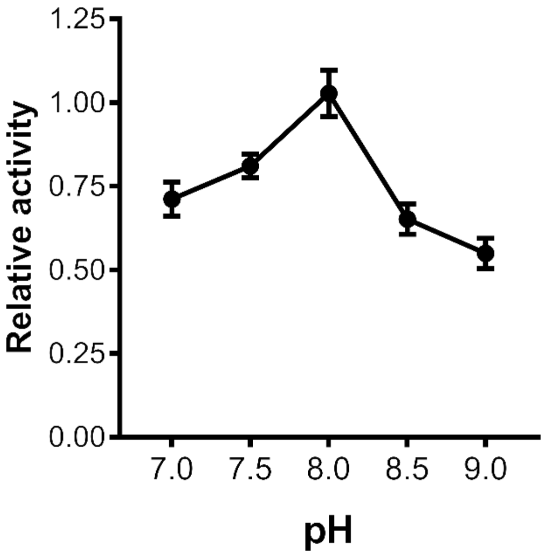

Supplementary Figure 2. The effect of the different pH buffers on the activity of TfS3GT2 with diosgenin using UDP-glucose as the donor. The Tris-HCl buffers with the range of pH 7.0-9.0 were utilized for this purpose. The relative enzyme activity was measured by quantifying the HPLC peak area of the glycosylated product (*i.e.*, trillin). The activity at the pH 8.0 was considered as a value of 100%, and the activities at the other pH values were accordingly calculated. All data are expressed as mean ± standard (SD).

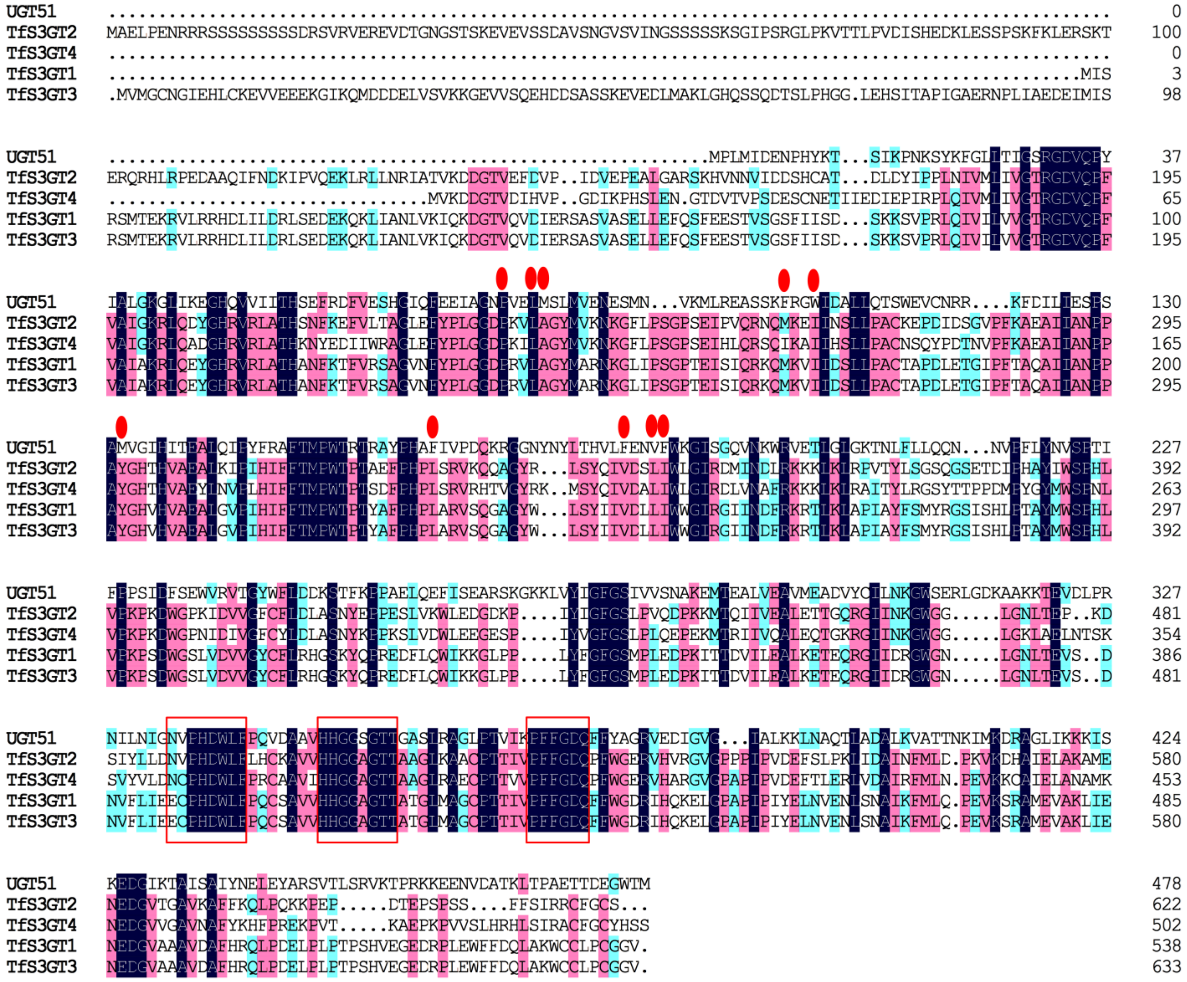

Supplementary Figure 3. Amino acid alignment of the four TfS3GT candidates and UGT51. The residues responsible for binding of UDP-glucose in the UGT51 cavity are indicated by square frame in red colors. The key residues surrounding the acceptor binding pocket are shown by red dots.

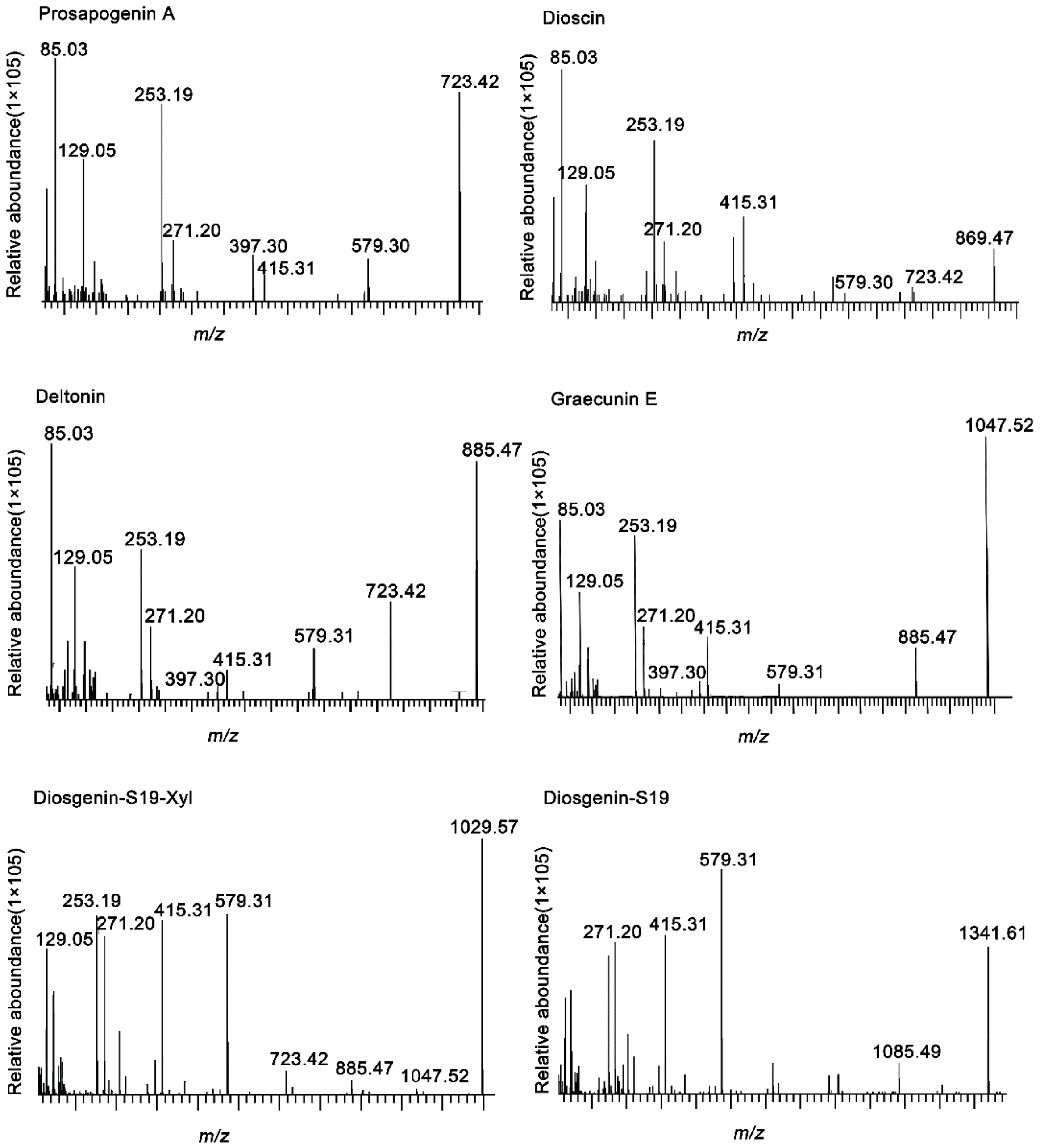

Supplementary Figure 4. Mass spectra of the targeted steroidal saponins extracted from the *T. foenum-graecum* transgenic hairy roots by the LC-MS in a positive mode.
